# Preoperative Botulinum Toxin Type A Use in Giant Inguinal Hernia Repair: A Scoping Review

**DOI:** 10.3390/medsci14010135

**Published:** 2026-03-13

**Authors:** Agostino Fernicola, Luigi Ricciardelli, Alessio Cece, Floriana Porcaro, Domenico Parmeggiani, Michele Santangelo, Gennaro Quarto

**Affiliations:** 1 Division of Endoscopic Surgery, Department of Clinical Medicine and Surgery, “Federico II” University of Naples, Via Pansini 5, 80131 Naples, Italygquarto@unina.it (G.Q.); 2 Unit of Emergency Surgery, Dei Colli Hospital, CTO Hospital, 80131 Naples, Italy; ricciardelliluigi@gmail.com; 3 Department of Integrated Activities in Surgery, Orthopedy and Hepato-Gastroenterology, University of Campania “Luigi Vanvitelli”, 80138 Naples, Italy; alessio.cece@unicampania.it (A.C.); domenico.parmeggiani@unicampania.it (D.P.); 4 Unit of Emergency Surgery, Department of Advanced Biomedical Sciences, Federico II University, 80131 Naples, Italy

**Keywords:** abdominal wall compliance, botulinum toxin A, giant inguinal hernia, hernia surgery, loss of domain

## Abstract

**Purpose**: Giant inguinal hernias (GIHs) are rare and technically demanding conditions, associated with loss of domain and abdominal wall compliance. Preoperative botulinum toxin type A (BtxA) has been increasingly used in complex ventral hernia repair to facilitate abdominal wall relaxation; however, its role in GIHs surgery remains poorly defined. This scoping review aimed to map the literature on preoperative BtxA use in GIHs repair, focusing on technical protocols, patient selection, and areas of variability. **Methods**: A scoping review was conducted in accordance with PRISMA-ScR guidelines. MEDLINE, Embase, Web of Science, and Scopus were searched from inception to 10 October 2025. Studies reporting preoperative BtxA administration in adult patients undergoing GIHs repair were included. Data were extracted descriptively and synthesized narratively. **Results**: Seven observational and non-comparative studies published between 2019–2025 were included, comprising a total of 16 patients. Substantial heterogeneity was observed in BtxA protocols, with total doses ranging from 100 to 450 units, injection timing between 2 and 8 weeks preoperatively, and injection sites varying from 6 to 18. In several reports, BtxA was used as part of a multimodal preoperative strategy including progressive pneumoperitoneum. All studies targeted the lateral abdominal wall musculature, employed imaging guidance, and performed bilateral injections. Patient selection criteria and outcome reporting were inconsistent. **Conclusions**: Preoperative BtxA use in GIHs repair is limited and heterogeneous. No standardized protocol can be identified. Further anatomically focused and systematically designed studies are required to clarify the role of BtxA and to establish standardized preoperative protocols for this challenging surgical condition.

## 1. Introduction

Giant inguinal hernias (GIHs) represent a rare and particularly demanding condition within the spectrum of abdominal wall pathology [1,2,3,4]. Unlike standard inguinal hernias, they are typically the result of a long-standing disease process, often evolving over many years and leading to profound anatomical distortion [1,2,3,5,6]. In these cases, herniation frequently extends into the scrotum, with progressive displacement of abdominal viscera and, in a substantial proportion of patients, the development of true loss of domain [1,2,3]. In fact, in most clinical scenarios the “giant” phenotype corresponds to a true inguinoscrotal hernia, as the scrotum provides the anatomical space necessary to accommodate progressively displaced abdominal viscera [1,2,3].

Surgical repair is therefore associated with unique technical challenges, including difficult reduction, excessive tension on the abdominal wall, respiratory compromise following visceral reintegration, and an increased risk of postoperative morbidity [1,2,3,7].

Despite their clinical relevance, GIHs remain poorly defined in the literature [1,2,3,7]. The lack of a standardized definition represents not only a descriptive limitation but also a methodological issue, as it hampers reproducibility, comparison across studies, and the development of objective criteria for patient selection and preoperative optimization strategies. No universally accepted classification exists, and the term is applied inconsistently across reports, often relying on descriptive anatomical criteria rather than standardized measurements [1,2,3,7]. In most published studies, the designation “giant” refers to inguinal or inguinoscrotal hernias extending well below the inguinal ligament and associated with significant chronicity and functional impairment [7]. For the purposes of this review, the term giant inguinal hernia (GIH) is used throughout to describe this shared clinical phenotype, without attempting retrospective reclassification based on arbitrary size thresholds.

In recent years, considerable interest has focused on preoperative strategies aimed at optimizing abdominal wall compliance in complex hernia repair [8,9,10,11,12,13,14,15]. Among these, preoperative injections of BtxA have emerged as a pharmacological adjunct capable of inducing temporary chemical paralysis of the lateral abdominal wall musculature [8,9,10,11,12,13,16,17]. By targeting the external oblique, internal oblique, and transversus abdominis muscles, BtxA promotes muscle elongation and reduces resting tone, thereby increasing abdominal wall compliance [1,3,16,17]. This approach, often referred to as chemical component separation, has been extensively investigated in patients with large ventral and incisional hernias, particularly in the presence of loss of domain, where it has been associated with improved feasibility of primary fascial closure and reduced reliance on surgical component separation techniques [8,9,10,11,12,13,15,18,19].

However, the anatomical and biomechanical context of GIH differs fundamentally from that of ventral or midline defects [8,9,10]. In inguinal hernias, the surgical challenge extends beyond midline approximation and includes the reintegration of chronically herniated viscera, adaptation of a contracted abdominal cavity, and management of tension across the lower and lateral abdominal wall [18,19]. As a result, protocols developed for ventral hernia repair cannot be directly extrapolated to the inguinal region. Nevertheless, this distinction is rarely acknowledged in the literature.

Notably, despite the growing body of evidence supporting the use of preoperative BtxA in abdominal wall reconstruction, GIHs are systematically excluded from most comparative studies, large observational cohorts, and systematic reviews. Recent meta-analyses have focused exclusively on ventral and incisional hernias, often explicitly excluding inguinal and inguinoscrotal defects from their analyses [8,9,10,11,12,13]. Consequently, evidence regarding the use of BtxA in GIH repair remains fragmented and is limited to isolated case reports and very small case series.

Across these reports, substantial heterogeneity exists in virtually every aspect of BtxA administration, including total dose, dilution and injected volume, timing relative to surgery, target muscles, imaging guidance, number and distribution of injection sites, and the use of adjunctive techniques such as progressive pneumoperitoneum.

Given the paucity and heterogeneity of the available evidence, a scoping review represents the most appropriate methodological approach to address this gap. Rather than evaluating efficacy or comparative outcomes, the primary objective of this review is to map the existing literature on preoperative BtxA use in GIH repair. Specifically, this scoping review aims to describe how BtxA has been administered, to summarize patient selection criteria, and to identify areas of technical variability and lack of standardization. By providing a structured overview of current practice, this work seeks to inform clinical decision-making and to support the design of future studies in this underexplored and clinically challenging field.

## 2. Materials and Methods

This scoping review was conducted in accordance with the Preferred Reporting Items for Systematic Reviews and Meta-Analyses extension for Scoping Reviews guidelines. The methodological approach was selected a priori to allow a comprehensive mapping of the existing literature on preoperative BtxA use in GIH repair, acknowledging the limited number of studies and the heterogeneity of available evidence. A scoping review methodology was selected because the available literature on the use of BtxA in GIH repair is limited and highly heterogeneous, consisting predominantly of case reports and small case series. In such situations, a scoping review is considered more appropriate than a systematic review, as it allows mapping the available evidence, identifying knowledge gaps, and providing an overview of the existing literature without attempting statistical synthesis.

The review protocol was prospectively registered on the Open Science Framework on 14 December 2025 (registration DOI: 10.17605/OSF.IO/XPWN6), prior to study selection and data extraction. The literature search was completed on 10 October 2025, and included studies available by that date, including online-ahead-of-print publications.

### 2.1. Review Questions and Objectives

The primary objective of this scoping review was to characterize how BtxA has been used preoperatively in the surgical management of GIHs. Rather than assessing effectiveness or comparative outcomes, the review sought to describe technical aspects of administration, including dosing strategies, timing relative to surgery, target muscles, injection technique, and use of imaging guidance. Secondary objectives included identifying patient selection criteria reported in the literature and highlighting areas of variability and lack of standardization across published studies.

### 2.2. Eligibility Criteria

Eligibility criteria were defined according to the Population, Concept, and Context framework. The population of interest included adult patients undergoing surgical repair of giant inguinal or inguinoscrotal hernias. Studies were included when the hernia was explicitly described as giant or when the clinical description clearly indicated a large, long-standing inguinal or inguinoscrotal hernia, often associated with loss of domain. No attempt was made to retrospectively reclassify hernias based on size thresholds, given the absence of standardized definitions in the literature.

The concept of interest was the preoperative use of BtxA as an adjunct to surgical repair. Studies were eligible if BtxA was administered prior to surgery with the intent of facilitating hernia reduction, improving abdominal wall compliance, or optimizing operative feasibility. Studies were included regardless of the use of additional preoperative adjuncts, such as progressive pneumoperitoneum, provided that botulinum toxin A administration in giant inguinal hernia repair was clearly reported. Across the included studies, patient selection for preoperative BtxA administration was primarily based on clinical judgment rather than standardized criteria. Commonly reported indications included long-standing giant inguinal or inguinoscrotal hernias with anticipated difficulty of visceral reduction, presence or suspicion of loss of domain, and perceived risk of excessive abdominal wall tension or postoperative respiratory compromise following reintegration. In most reports, these factors were assessed qualitatively, without predefined volumetric thresholds or formal risk stratification tools.

Reports in which BtxA was used postoperatively or for indications unrelated to hernia repair were excluded.

The context was limited to clinical surgical practice. Case reports, case series, and observational studies were eligible for inclusion. Systematic reviews, narrative reviews, conference abstracts, animal studies, pediatric studies, and reports focusing exclusively on ventral, incisional, diaphragmatic, or traumatic hernias were excluded. Studies in which inguinal hernia cases were embedded within ventral hernia cohorts without separate reporting were also excluded.

### 2.3. Information Sources and Search Strategy

A comprehensive literature search was performed in multiple electronic databases, including MEDLINE, Embase, Web of Science and Scopus, from inception to 10 October 2025. The search strategy combined controlled vocabulary terms and free-text keywords related to BtxA, inguinal hernia, giant hernia, inguinoscrotal hernia, and loss of domain. Reference lists of included studies and relevant reviews were manually screened to identify additional eligible reports. The electronic search strategy combined controlled vocabulary terms and free-text keywords related to BtxA and inguinal hernia, using the following core query adapted to each database: (botulinum toxin A OR botox OR chemodenervation) AND (inguinal hernia OR inguinoscrotal hernia OR groin hernia) AND (giant OR massive OR loss of domain). For MEDLINE, the full search string was: (“botulinum toxin A” OR botox OR chemodenervation) AND (“inguinal hernia” OR inguinoscrotal hernia OR groin hernia) AND (giant OR massive OR “loss of domain”). No language restrictions were applied during the literature search.

### 2.4. Study Selection

**Figure 1:**
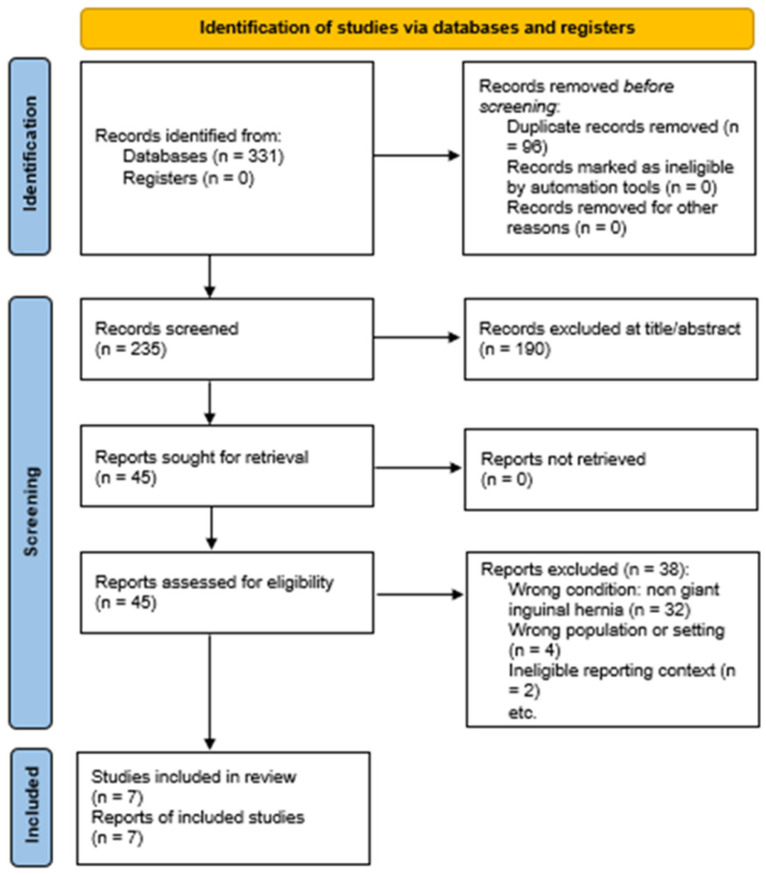
PRISMA 2020 flow diagram adapted for scoping reviews (PRISMA-ScR) of study selection [20].

All retrieved records were imported into reference management software (Mendeley Reference Manager: the version is 2.143.0), and duplicates were removed prior to screening. Titles and abstracts were screened independently by two reviewers to assess potential eligibility. Full-text articles were obtained for all studies deemed potentially relevant. Full-text screening was performed independently by the same reviewers, with discrepancies resolved through discussion and consensus. Reasons for exclusion at the full-text stage were documented in detail and are reported in the PRISMA flow diagram (Figure 1).

### 2.5. Data Extraction and Charting

Data extraction was performed using a standardized data charting form developed specifically for this review. The form was pilot tested on a subset of included studies and refined iteratively. Extracted data included study characteristics, patient demographics, hernia description, and details of surgical approach. Particular attention was given to technical aspects of BtxA administration, including formulation, total dose, dilution, volume injected, number and distribution of injection sites, target muscles, use of imaging guidance, laterality of injections, and timing relative to surgery. When BtxA was used in combination with other preoperative adjuncts, such as progressive pneumoperitoneum, this was recorded explicitly. In addition, information on patient selection criteria and reported clinical rationale for BtxA use was extracted. Elements that were inconsistently reported or not reported at all were noted to identify gaps in standardization across studies.

### 2.6. Data Synthesis

Given the descriptive nature of the available evidence, data were synthesized narratively. Findings are presented as a structured summary of study characteristics and technical protocols, with emphasis on identifying patterns, ranges, and areas of heterogeneity. No formal assessment of methodological quality or risk of bias was performed, in keeping with the objectives of a scoping review and the predominance of non-comparative study designs. Quantitative synthesis was not attempted.

## 3. Results

### 3.1. Study Selection

The literature search yielded a substantial number of records, reflecting the broad use of BtxA in abdominal wall surgery. After removal of duplicates, titles and abstracts were screened for relevance. The majority of studies were excluded at this stage because they focused exclusively on ventral or incisional hernias, pediatric populations, traumatic defects, or non-clinical settings. Full-text assessment was performed for a limited subset of articles in which inguinal hernia involvement was either explicitly mentioned or potentially implied.

Following full-text review, seven studies met the predefined eligibility criteria and were included in the final synthesis. All included studies specifically addressed the use of preoperative BtxA in the context of giant inguinal or inguinoscrotal hernia repair and provided sufficient technical detail to allow data extraction. Reasons for exclusion at the full-text stage were most related to the absence of inguinal-specific data or the inclusion of inguinal hernias as non-separable cases within ventral hernia cohorts. The study selection process is summarized in the PRISMA flow diagram.

### 3.2. Characteristics of Included Studies

The seven included studies were published between 2019 and 2025 and comprised a total of sixteen patients undergoing surgical repair of giant inguinal hernias. All studies were observational in nature. Six were case reports or small case series, and one was a prospective observational study.

Across all included reports, hernias were described as long-standing, large inguinal or inguinoscrotal defects, frequently extending well below the inguinal ligament and associated with marked anatomical distortion. These descriptions reflect a set of shared clinical characteristics across the included cases, including long-standing inguinoscrotal extension, substantial displacement of abdominal viscera, and clinical or radiological features suggestive of loss of domain. Loss of domain was explicitly reported in most cases, either on clinical grounds or through radiological assessment. Only one study applied a quantitative volumetric threshold to define loss of domain, whereas the remaining reports relied on descriptive or qualitative criteria.

Surgical approaches varied and included open inguinal repair as well as minimally invasive techniques. In several studies, preoperative BtxA was administered as part of a multimodal strategy that also included progressive pneumoperitoneum rather than as an isolated intervention. In four studies, BtxA administration was combined with progressive pneumoperitoneum as part of a multimodal preoperative strategy. In the remaining studies, BtxA was used as a standalone adjunct. Given the heterogeneity of surgical techniques and adjunctive measures, no attempt was made to compare operative outcomes across studies.

Overall, the seven included studies accounted for 16 patients, as detailed in Table 1.

### 3.3. Botulinum Toxin A Administration Protocols

Substantial variability was observed in BtxA administration protocols across the included studies (Table 2). All reports used BtxA, but the formulation and dosing strategies differed considerably. The total administered dose ranged from 100 to approximately 450 units. The lowest dose was reported in a prospective series in which BtxA was administered in conjunction with progressive pneumoperitoneum, whereas higher doses were used in isolated case reports and small series [15,18]. In several studies, the total dose was not explicitly stated but could be inferred from the reported dose per injection site and number of sites.

Timing of BtxA injections related to surgery also varied. In most studies, injections were performed between two and eight weeks prior to the planned operation, corresponding to the expected peak pharmacological effect [14,15,17,19,21]. In contrast, one study administered BtxA two to three weeks before surgery, coinciding with the initiation of progressive pneumoperitoneum [18]. No study directly compared different timing strategies or justified the chosen interval based on pharmacodynamic considerations.

All included studies targeted the lateral abdominal wall musculature [14,15,17,18,19,21]. Injections were consistently directed at the external oblique, internal oblique, and transversus abdominis muscles, although the depth and sequence of muscle infiltration were not uniformly described. None of the reports evaluated selective muscle paralysis or compared different combinations of target muscles. Instead, full-thickness lateral abdominal wall paralysis was applied empirically in all cases.

The number and distribution of injection sites varied markedly. Reported protocols ranged from six total injection sites to as many as eighteen, with all studies employing bilateral injections. Injection sites were typically distributed along the anterior or mid-axillary line, between the costal margin and the iliac crest. However, the rationale for the number of sites and their precise anatomical localization was rarely explained.

### 3.4. Imaging Guidance and Technical Aspects

Imaging guidance was explicitly reported in six of the seven included studies, with ultrasound guidance used in five studies and computed tomography guidance in one; one study did not clearly specify the imaging modality employed. Ultrasound guidance was the most reported technique and was used to identify muscle layers and guide needle placement in five studies [14,17,18,19,21]. In one report, injections were performed under computed tomography guidance by an interventional radiology team [15]. No study compared imaging modalities or assessed their impact on accuracy, safety, or clinical outcomes.

Details regarding dilution, injected volume per site, and needle technique were inconsistently reported. In some studies, dilution and volume were described in detail, allowing calculation of dose per muscle layer and per injection site [14,17,18]. In others, these parameters were either incompletely reported or omitted altogether, limiting reproducibility [15,19,21].

### 3.5. Patient Selection Criteria

Criteria for selecting patients for preoperative BtxA administration were variably described. All studies included patients with giant inguinal or inguinoscrotal hernias considered at high risk for difficult reduction or postoperative complications. Loss of domain, either clinically evident or radiologically confirmed, was the most frequently cited indication. One study employed a predefined volumetric threshold to guide patient selection, whereas the remaining reports relied on clinical judgment without explicit criteria [18].

No study provided standardized exclusion criteria or discussed patient factors that might contraindicate BtxA use. Reporting of adverse events related to BtxA administration was inconsistent across studies. Most reports did not systematically describe safety outcomes, and no study provided structured or standardized reporting of BtxA-related complications. Similarly, none reported formal assessment of neuromuscular conditions or previous exposure to BtxA as part of patient selection.

### 3.6. Areas of Non-Standardization

Across the included studies, lack of standardization emerged as a consistent finding. Considerable heterogeneity was observed in dosing, timing, number of injection sites, imaging guidance, and adjunctive preoperative strategies. Even when similar elements were used, such as bilateral injections or targeting of all three lateral abdominal wall muscles, the underlying rationale was seldom articulated. As a result, it was not possible to identify a shared or consensus protocol for BtxA administration in GIH repair. Key areas of heterogeneity and lack of standardization identified across the included studies are summarized in Table 3.

## 4. Discussion

This scoping review provides the first structured synthesis of the available literature on preoperative BtxA use in the GIHs repair, identifying seven observational studies and highlighting substantial heterogeneity in patient selection and technical protocols [14,15,16,17,18,19,20]. The principal finding is not the identification of a shared protocol or consistent strategy, but rather the striking paucity and heterogeneity of the evidence. Despite an extensive body of literature supporting BtxA as an adjunct in ventral and incisional hernia repair, its application in GIHs remains confined to a small number of descriptive reports, with substantial variation in technique, patient selection, and perioperative strategy.

The limited number of included studies should not be interpreted as a failure of the search strategy or a weakness of this review. On the contrary, it reflects a genuine gap in the literature. Large observational cohorts, comparative studies, and systematic reviews addressing BtxA in abdominal wall reconstruction consistently exclude inguinal hernias, often explicitly. This exclusion likely stems from the perception that inguinal hernias, even when large, represent a fundamentally different problem from ventral defects [8,9,10,11,12]. However, in the context of GIHs with loss of domain, this distinction becomes less clear, as the physiological challenges of visceral reintegration and abdominal compartment adaptation overlap substantially with those encountered in massive ventral hernias [3,8,9,10,11,12]. Nevertheless, current European Hernia Society guidelines emphasize that abdominal wall biomechanics and reconstructive principles differ substantially between midline and non-midline defects [22]. This conceptual distinction supports the need for caution when extrapolating preoperative preparation strategies, such as botulinum toxin administration, from ventral to inguinal hernia repair [13,22,23].

The absence of inguinal-specific evidence synthesis therefore represents an unmet clinical and research need. At the same time, giant inguinal hernias pose unique biomechanical challenges that are not fully captured by ventral hernia paradigms [24,25,26,27]. The asymmetric displacement of viscera into the inguinoscrotal compartment, the chronic deformation of the lower abdominal wall, and the altered force vectors acting on the lateral musculature differentiate GIHs from midline defects [3,8,10,12]. These features may partially explain why protocols developed for ventral hernia repair cannot be directly translated to the inguinal region and underscore the need for anatomically tailored preoperative strategies.

Although this scoping review was not designed to assess clinical effectiveness, several reports provided descriptive observations regarding the perceived clinical impact of preoperative BtxA administration. Authors frequently cited facilitated reduction in chronically herniated viscera, improved abdominal wall compliance, and subjective reduction in intraoperative tension as key reasons for BtxA use. In studies combining BtxA with progressive pneumoperitoneum, the intervention was often described as part of a strategy to mitigate the risk of postoperative abdominal compartment syndrome or respiratory compromise [15,16,18,21]. However, these observations were largely anecdotal and were not supported by standardized outcome measures, comparative analyses, or objective physiological assessments. Importantly, none of the included studies formally evaluated potential adverse effects or systematically assessed safety outcomes related to BtxA administration in this setting. Reported complications were inconsistently documented, and the absence of standardized follow-up protocols limits interpretation of both benefits and risks. Although serious adverse events related to preoperative BtxA appear uncommon, recent reports in the broader abdominal wall reconstruction literature have described rare cardiopulmonary complications, particularly in elderly patients with significant comorbidities [28]. The absence of standardized safety reporting in GIH-specific studies therefore represents a critical gap, especially given the fragile physiological reserve often observed in patients with long-standing giant hernias.

Total doses varied more than fourfold across studies, ranging from 100 to approximately 450 units, without a clear rationale linking dose selection to patient characteristics, hernia size, or surgical approach. Notably, one recent case report incorporated detailed pre- and post-treatment computed tomography to quantitatively assess changes in abdominal cavity volume and lateral muscle geometry following BtxA administration [17]. While imaging was not used to guide injections, this volumetric approach provides objective insight into the anatomical effects of BtxA and illustrates how imaging-based metrics may help refine patient selection and optimize timing in future studies [17].

Timing of injection relative to surgery also differed, with intervals spanning from two to eight weeks, despite the known pharmacodynamics of BtxA. Similarly, although all studies targeted the lateral abdominal wall musculature, none explored selective muscle paralysis or justified the routine inclusion of all three muscle layers. These observations suggest that current practice is largely empirical and extrapolated from ventral hernia protocols rather than grounded in inguinal-specific evidence [9,11].

The use of imaging guidance further illustrates this variability. While ultrasound guidance was most commonly employed, one study relied on computed tomography guidance, reflecting differences in institutional expertise rather than evidence-based choice [15]. No study compared imaging modalities or assessed their impact on safety or efficacy, as described in ventral hernias [29,30,31,32]. Details regarding dilution, injected volume, and needle technique were frequently incomplete, limiting reproducibility and highlighting the need for more rigorous reporting standards in future studies.

Patient selection criteria were similarly inconsistent. Although loss of domain was a recurring theme, only one study employed a quantitative volumetric threshold to guide inclusion [18]. In the remaining reports, selection was based on clinical judgment, often without explicit criteria. This lack of standardization complicates interpretation of outcomes and hinders comparison across studies. It also raises the question of whether BtxA should be reserved for a narrowly defined subset of patients with GIHs or considered more broadly in selected cases at high risk of difficult reduction or postoperative complications.

An important consideration emerging from this review is the frequent use of BtxA as part of a multimodal preoperative strategy rather than as an isolated intervention. In several studies, BtxA was combined with progressive pneumoperitoneum and advanced minimally invasive techniques [15,16,18,21]. While this integrated approach reflects real-world practice in highly specialized centers, it makes it difficult to isolate the specific contribution of BtxA to surgical feasibility or outcomes. Future studies should aim to clarify whether BtxA provides incremental benefit beyond other preoperative adjuncts or whether its role is primarily synergistic.

From a clinical perspective, preoperative BtxA has been used almost exclusively in highly selected patients with giant inguinal hernias considered at increased risk of difficult visceral reduction or postoperative physiological compromise. In the absence of standardized patient selection criteria, its use appears to be driven primarily by individual surgeon judgment rather than predefined anatomical or volumetric thresholds. Although all reported protocols targeted the lateral abdominal wall musculature, substantial variability in dosing, timing, and injection technique was observed, reflecting the lack of inguinal-specific technical frameworks. For context, the maximum authorized doses of botulinum toxin A vary depending on the formulation and clinical indication but generally range up to approximately 300–400 units per treatment session for most therapeutic applications. The doses reported in the reviewed studies fall within this range, supporting the overall safety profile of BtxA administration in the preoperative setting.

These findings suggest that preoperative BtxA should currently be regarded as an adjunctive strategy reserved for selected cases in specialized centers, rather than a routinely applicable preoperative intervention. This interpretation is also consistent with the fact that many GIHs can be repaired without adjunctive preoperative techniques in experienced hands. Moreover, the very small number of reported inguinal cases contrasts with the much broader literature on ventral and incisional hernias, where the role of preoperative BtxA is more established. These considerations suggest that BtxA is currently better supported in midline and incisional abdominal wall reconstruction than in inguinal hernia surgery, where it should remain a selective option for carefully chosen cases, particularly in the presence of loss of domain.

This review has limitations that warrant acknowledgment. The evidence base is small and consists exclusively of non-comparative studies, precluding any assessment of effectiveness or safety beyond descriptive observations. Reporting quality varied across studies, and missing data limited the ability to fully reconstruct technical protocols in some cases. However, these limitations are inherent to the available literature and underscore, rather than diminish, the relevance of this scoping review.

The findings of this review have several implications for clinical practice and future research. From a clinical perspective, they highlight the need for caution when extrapolating ventral hernia protocols to the inguinal region and emphasize the importance of individualized decision-making in complex cases. Until higher-quality evidence becomes available, the use of preoperative BtxA in GIH repair should be considered on a case-by-case basis within specialized centers. From a research standpoint, they underscore the need for standardized reporting of BtxA administration in GIH repair, including clear definitions, detailed technical descriptions, and transparent patient selection criteria. Even small prospective series, if methodologically rigorous and consistently reported, could substantially advance understanding in this field.

Although the number of reported patients remains very small and does not allow statistically robust conclusions, the available evidence suggests that preoperative BTA may offer clinically relevant advantages. Several studies reported an increase in abdominal cavity volume and elongation of the lateral abdominal wall muscles after toxin administration, facilitating visceral reintegration and tension-free repair. Moreover, the reviewed cases consistently described minimal complications related to BTA injection, supporting its potential role as a safe adjunctive strategy in selected patients with giant inguinal hernias.

In conclusion, preoperative BtxA use in GIH repair remains an emerging and incompletely defined practice. The existing evidence is limited, heterogeneous, and largely descriptive, reflecting a significant gap in the literature. By mapping current practice and identifying areas of non-standardization, this scoping review provides a foundation for future studies aimed at defining the role of BtxA in this challenging clinical setting.

## 5. Conclusions

The use of preoperative BtxA in GIH repair is supported by very limited and heterogeneous evidence, confined to small descriptive studies with substantial variability in dosing, timing, injection technique, and patient selection, while all studies consistently targeted the three lateral abdominal wall muscles without providing a standardized rationale for muscle selection or evaluating selective muscle paralysis strategies. No standardized protocol can currently be identified. This scoping review highlights a significant gap in literature and underscores the need for anatomically specific, systematically reported studies to better define the role of BtxA in GIH management.

## Figures and Tables

**Table 1:** Characteristics of included studies.

Author (Year)	Country	Study Design	Patients (n)	Hernia Type	Loss of Domain Assessment	Surgical Approach	Progressive Pneumoperitoneum	Mesh Used
Tang et al. (2019) [18]	China	Prospective observational study	8	Giant inguinoscrotal	Radiological (VIH/VAC ratio)	Laparoscopic TAPP	Yes	Yes
Sanford et al. (2019) [15]	United States	Case series	2	Giant bilateral inguinoscrotal	Clinical and radiological	Minimally invasive (eTEP/TAR)	Yes	Yes
Gonzalo et al. (2019) [21]	Spain	Case report	1	Giant inguinal	Clinical and radiological	Open inguinal repair with scrotoplasty	Yes	Yes
Menenakos et al. (2020) [16]	Germany	Case report	1	Giant bilateral inguinoscrotal	Clinical assessment	Open preperitoneal (Stoppa)	Yes	Yes
Avellana et al. (2020) [19]	Spain	Case report	1	Giant inguinoscrotal	Clinical assessment	Open inguinal repair	No	Yes
Huerta et al. (2024) [14]	Spain	Case series	2	Giant inguinoscrotal	Clinical assessment	Open inguinal repair	No	Yes
Zamkowski et al. (2025) [17]	Poland	Case report	1	Giant scrotal inguinal	Radiological (Tanaka index)	Open inguinal repair (Lichtenstein)	No	Yes

Legend: This table summarizes the main characteristics of studies included in the scoping review, including study design, patient population, hernia phenotype, assessment of loss of domain, surgical approach, and use of progressive pneumoperitoneum. Abbreviations: eTEP, extended totally extraperitoneal approach; TAR, transversus abdominis release; TAPP, transabdominal preperitoneal repair; VIH/VAC, hernia sac volume to abdominal cavity volume ratio.

**Table 2:** Preoperative BtxA administration protocols in GIH repair (studies with sufficient technical detail).

Author (Year)	BtxA Formulation	Total Dose (Units)	Dilution/Volume	Target Muscles	Imaging Guidance	Injection Sites (Total)	Bilateral Injections	Timing Before Surgery
Sanford et al. (2019) [15]	BtxA (not specified)	~450	Not reported	EO, IO, TA	CT	18 (9 per side)	Yes	4 weeks
Tang et al. (2019) [18]	Botox^®^ (onabotulinumtoxinA)	100	2 IU/mL	EO, IO, TA	Ultrasound	6 (bilateral)	Yes	2–3 weeks
Gonzalo et al. (2019) [21]	BtxA (not specified)	Not reported	Not reported	EO, IO, TA	Ultrasound	10 (bilateral)	Yes	4 weeks
Avellana et al. (2020) [19]	Botox^®^ (onabotulinumtoxinA)	400	Not reported	EO, IO, TA	Ultrasound	6–8 (bilateral)	Yes	4–6 weeks
Huerta et al. (2024) [14]	Botox^®^ (onabotulinumtoxinA)	200	2 IU/mL	EO, IO, TA	Ultrasound	6 (3 per side)	Yes	4 weeks
Zamkowski et al. (2025) [17]	Dysport^®^ (abobotulinumtoxinA)	300	300 IU/150 mL saline	EO, IO, TA	Ultrasound	6 (3 per side)	Yes	8 weeks

Legend: This table details the technical characteristics of preoperative BtxA administration reported in studies included in the scoping review. Only studies reporting sufficient technical details for protocol reconstruction are shown. Substantial heterogeneity is observed across all domains, including total dose, dilution, number of injection sites, imaging guidance, and timing relative to surgery. Abbreviations: BtxA, botulinum toxin type A; EO, external oblique muscle; IO, internal oblique muscle; TA, transversus abdominis muscle; CT, computed tomography; IU: International Units.

**Table 3:** Areas of non-standardization in preoperative BtxA use for GIH repair.

Domain	Observed Variability
Definition of GIH	No standardized definition; classification based on descriptive anatomical criteria and clinical judgment
Patient selection criteria	Qualitative assessment in most studies; quantitative volumetric threshold reported in only one study
Total BtxA dose	Wide range from 100 to approximately 450 units, with no dose–response rationale
Timing of injection	Intervals ranging from 2 to 8 weeks before surgery
Target muscles	External oblique, internal oblique, and transversus abdominis targeted in all studies, without evaluation of selective paralysis
Imaging guidance	Ultrasound guidance in most studies; computed tomography guidance in one study
Number of injection sites	Highly variable, ranging from 6 to 18 total sites
Injection technique details	Inconsistent reporting of dilution, injected volume, and needle approach
Use of adjunctive strategies	BtxA used alone or in combination with progressive pneumoperitoneum
Outcome reporting	Heterogeneous and largely descriptive, with no standardized outcome measures

Abbreviations: BtxA, botulinum toxin type A.

## Data Availability

No new data were created.

## Abbreviations

The following abbreviations are used in this manuscript:

GIHs	Giant inguinal hernias
BtxA	Botulinum toxin type A
GIH	Giant inguinal hernia
